# Accurate Extraction of Phenotypic Parameters for DUS Testing of *Oudemansiella raphanipes* Based on Deep Learning

**DOI:** 10.3390/jof12100778

**Published:** 2026-10-16

**Authors:** Tao Yuan, Dong Hu, Mengyuan Xu, Minglu Tian, Hailong Yu, Linyi Li

**Affiliations:** 1 Institute of Agricultural Science and Technology Information, Shanghai Academy of Agricultural Sciences, Shanghai 201403, China; yuantao@saas.sh.cn (T.Y.); 20220801@saas.sh.cn (D.H.); m18845795197@163.com (M.X.); tianminglu@saas.sh.cn (M.T.); 2 Institute of Edible Fungi, Shanghai Academy of Agricultural Sciences, National Engineering Research Center of Edible Fungi, Shanghai 201403, China

**Keywords:** *Oudemansiella raphanipes*, DUS testing, deep learning, instance segmentation, YOLOv11s, phenotypic parameter extraction, computer vision

## Abstract

To enable the rapid and accurate acquisition of phenotypic parameters during distinctness, uniformity, and stability (DUS) testing of edible mushroom varieties, and to address the low efficiency, strong subjectivity, and insufficient standardization of conventional manual measurement methods, this study used the fruiting bodies of *Oudemansiella raphanipes* as the research object and proposed an automatic phenotypic parameter extraction method based on deep learning instance segmentation. An instance-segmentation dataset containing 473 *O. raphanipes* images and 1419 annotated objects was constructed, and the YOLOv11s-seg model was improved by introducing the lightweight feature-extraction network StarNet_s050 to construct the YOLOv11s-star-seg model, thereby improving model inference efficiency and meeting practical application requirements. Under standardized imaging conditions, pileus and stipe regions were obtained from the instance-segmentation results, and image-scale calibration was combined to automatically extract DUS phenotypic parameters, including pileus diameter, pileus context thickness, stipe length, and stipe diameter, while quantitatively describing stipe shape. The experimental results showed that the YOLOv11s-star-seg model performed well in the instance segmentation of *O. raphanipes*, achieving an mAP50 of 99.1%, an F1 value of 98.9%, and an mIoU of 97.86%; compared with the YOLOv11s-seg model, the number of parameters was reduced by 39% and the inference time was reduced by 27%, thereby achieving model lightweighting while maintaining high segmentation accuracy. The automatically extracted results showed high consistency with the manual measurements; the coefficients of determination (R^2^) from linear regression fitting for stipe length, stipe diameter, and pileus diameter all exceeded 0.96, indicating that the method can effectively replace part of the manual measurement process. The results can provide technical support for the digitalization of DUS testing of *O. raphanipes*, intelligent breeding of edible mushrooms, and accurate phenotypic identification.

## 1. Introduction

*Oudemansiella raphanipes*, commonly marketed in China as “black-skinned termitomyces”, is a rare edible and medicinal fungus in the genus *Oudemansiella*, family Physalacriaceae, order Agaricales. Studies have shown that ousenine (Oudemansiella ketone) contained in *O. raphanipes* has an antihypertensive effect, while *O. raphanipes* polysaccharides have effects including immune regulation, blood glucose reduction, blood lipid reduction, antioxidation, and inhibition of tumor cell growth [1].

As important agricultural products with both nutritional and economic value, edible mushrooms require phenotypic parameter measurement, growth status monitoring, quality grading, and species identification as important technical support for efficient breeding, industrialized production, and automated harvesting. Conventional manual phenotypic measurement has disadvantages such as high labor intensity, substantial time and effort requirements, strong subjectivity, and inconsistent standards, making it difficult to meet the needs of modern production. In recent years, the rapid development of computer-vision technology has provided diverse solutions for edible mushroom phenotypic measurement and intelligent detection [2,3]; related studies have covered multiple species, including shiitake mushrooms, button mushrooms, enoki mushrooms, and mini oyster mushrooms, and have formed several application directions, including phenotypic extraction for breeding, growth status recognition, quality grading, and classification and identification [4,5].

Phenotypic parameter extraction for edible mushroom breeding can be divided into mycelial phenotypic parameter extraction and fruiting body phenotypic parameter extraction. For mycelial phenotype extraction, the main research directions include the extraction and definition of mycelial phenotypic data such as colony perimeter, colony radius, colony area, colony coverage, and mycelial growth rate [6,7], the development of mycelial phenotype extraction software and integrated imaging equipment [8], and the improvement of measurement methods [9]. For phenotypic extraction from edible mushroom fruiting bodies, the main research directions include extraction of phenotypic information such as pileus shape, area, attachment position, and color, as well as stipe length, width, and color, in enoki mushroom fruiting bodies [10]; accurate segmentation of the pileus, stipe, and lamellae of shiitake mushrooms [11]; and simultaneous measurement of multiple fruiting bodies in single images containing multiple targets and different mushroom species [12].

Monitoring the developmental period and recognizing the growth status of edible mushroom fruiting bodies are prerequisites and key elements for automated harvesting, with emphasis on addressing occlusion, false detection, and discrimination among multiple growth stages. To address the segmentation of adherent mushrooms in complex backgrounds, Chen et al. [13] used visible-light images and proposed a segmentation and recognition algorithm for Agaricus bisporus based on watershed transformation; test results showed that the algorithm outperformed the existing circular Hough transform. Image-processing technology performs well and is relatively simple and efficient for mushroom images acquired under comparatively stable imaging conditions; however, when processing images from complex environments or variable acquisition conditions, image segmentation thresholds or segmentation algorithms must be repeatedly modified, demonstrating poor generalization and limiting its application. Therefore, deep learning-based image-segmentation and recognition methods have gradually become the current mainstream technology. To detect the growth status of industrially cultivated straw mushrooms, Zha et al. [14] collected images of straw mushrooms under different cultivation modes and growth periods, abnormal straw mushrooms, contamination of cultivation substrates by miscellaneous fungi, and other conditions as the original image dataset. The 895 collected original images were divided into seven categories: growth-stage, mature-stage, and opened-pileus straw mushrooms; straw mushrooms infected by miscellaneous fungi; navel-shaped mushrooms; ink-cap mushrooms; and surface infection of the cultivation substrate. The test results showed that the model achieved a mean precision of 83.7%. More studies have focused on improving YOLO models, for example, by improving YOLO series algorithms to automatically obtain mushroom counts [15] and pileus contours [16]; calculating pileus diameter, pileus circularity, pileus whiteness, and surface spots on the pileus [17]; recording pileus size, calculating mushroom growth rate, and evaluating mushroom harvesting time [18]; identifying mushroom positions, determining the bending direction of the pileus, and assigning robotic picking directions [19]; and addressing false and missed detections [20]. Some researchers have also considered that RGB images alone lack spatial depth perception capability [21]; integrating depth camera images can more accurately calculate the position, diameter, orientation angle, and inclination angle of individual mushrooms in the world coordinate system, thereby meeting the field requirements of mushroom-harvesting robots [22].

Quality grading focuses on the commercialization requirements of mushrooms. Huang et al. [23] used four characteristic variables—fractal dimension, relative displacement, pileus eccentricity, and stipe curvature—to identify malformed mini oyster mushrooms and provide technical support for their quality grading. In addition, pileus diameter [24], differences in pileus texture [], and differences among mushroom images under different freezing conditions [25] can also provide references for rapid mushroom quality identification and online automatic grading and sorting.

Research on the classification and identification of edible mushrooms mainly focuses on their fruiting bodies, although a small number of studies are based on mycelia. For example, Guan et al. [26] collected near-infrared spectral data from the mycelia of six mushroom varieties using a near-infrared spectrometer and constructed a mushroom-mycelium-variety recognition model using an eight-layer convolutional neural network (E-CNN), thereby achieving accurate and rapid recognition of edible mushroom mycelial varieties. In studies of edible mushroom fruiting body phenotypes, image-processing methods that integrate color space with morphological features and extract pileus texture features have achieved classification and recognition accuracies exceeding 90% [27,28]; by introducing deep learning classifiers, the classification and identification accuracy of mushroom fruiting bodies can exceed 95% [29,30].

In summary: (1) based on computer-vision technology, segmentation models and object detection algorithms can currently achieve the automatic extraction and measurement of multiple mycelial phenotypes, including colony perimeter, colony radius, colony area, colony coverage, and color, and mycelial growth rate can be calculated from differences in mycelial phenotypes at different times; (2) for edible mushroom fruiting bodies, multiple types of phenotypic information, including pileus shape, pileus area, pileus attachment position, pileus color, stipe length, stipe width, and stipe color, can be automatically extracted and calculated, and multiple fruiting bodies can be measured simultaneously in a single image; (3) the application of computer-vision technology to edible mushrooms is gradually shifting from image processing and machine learning algorithms to deep learning algorithms represented by the YOLO series, but because of differences among edible mushroom species and research purposes, no unified algorithm or model can acquire and identify all mushroom phenotypes; (4) in addition to the visible-light band, the near-infrared band has also been introduced as an auxiliary means of phenotype acquisition, and the inclusion of depth camera images can more accurately calculate the position, diameter, orientation angle, and inclination angle of individual mushrooms in the world coordinate system, providing data support for field harvesting by mushroom-harvesting robots; and (5) the vast majority of studies related to edible mushroom phenotypes remain at an early exploratory stage, and few have been genuinely integrated with industrial production applications.

This study used *O. raphanipes* fruiting bodies as the research object and, in combination with the practical application requirements of *O. raphanipes* DUS testing (Agricultural Industry Standard of the People’s Republic of China NY/T3715--2020) [31], proposed a deep learning-based method for *O. raphanipes* image segmentation and accurate acquisition of phenotypic parameters. The model was optimized for lightweighting; four indicators—stipe length, stipe diameter, pileus diameter, and pileus thickness—were obtained, and the results were compared with the original YOLOv11s-seg models. Finally, the qualitative descriptive indicator of *O. raphanipes* stipe shape was quantitatively defined, and stipe shape was classified based on the definition.

## 2. Materials and Methods

### 2.1. Data Acquisition and Processing

Data were collected at Shandong Century Smart Agriculture Technology Co., Ltd.(Jining, Shandong, China) *O. raphanipes* materials numbered 501, 504, 519, 525, 527, 528, and 531 were sampled in multiple cultivation batches under the same cultivation conditions (see Table 1 for the frequency distribution). Each individual fruiting body was imaged only once. Fruiting bodies at the mature developmental stage were selected for sampling, while samples with obvious mechanical damage or deformity were excluded to improve the representativeness and reliability of the dataset. Several fruiting bodies were randomly selected from each cultivation batch for image acquisition and phenotypic trait measurement to satisfy the requirements for sample size and replication in distinctness, uniformity, and stability (DUS) testing.

Images of *O. raphanipes* were acquired using a self-developed standardized phenotyping imaging box. The imaging box integrated an LED light source with a fixed color temperature of approximately 6500 K to reduce the effects of shadows and specular reflection on color measurement, a green background to enhance the contrast between the target and background, a standard ColorChecker (Calibrite, Grand Rapids, MI, USA) for image color correction, and a geometric calibration grid for pixel-to-actual-length conversion. An industrial camera was used for fixed-distance and fixed-focus imaging to ensure consistency in spatial resolution and imaging conditions among different cultivation batches. The original image resolution was 5472 × 3648 pixels.

**Figure 1:**
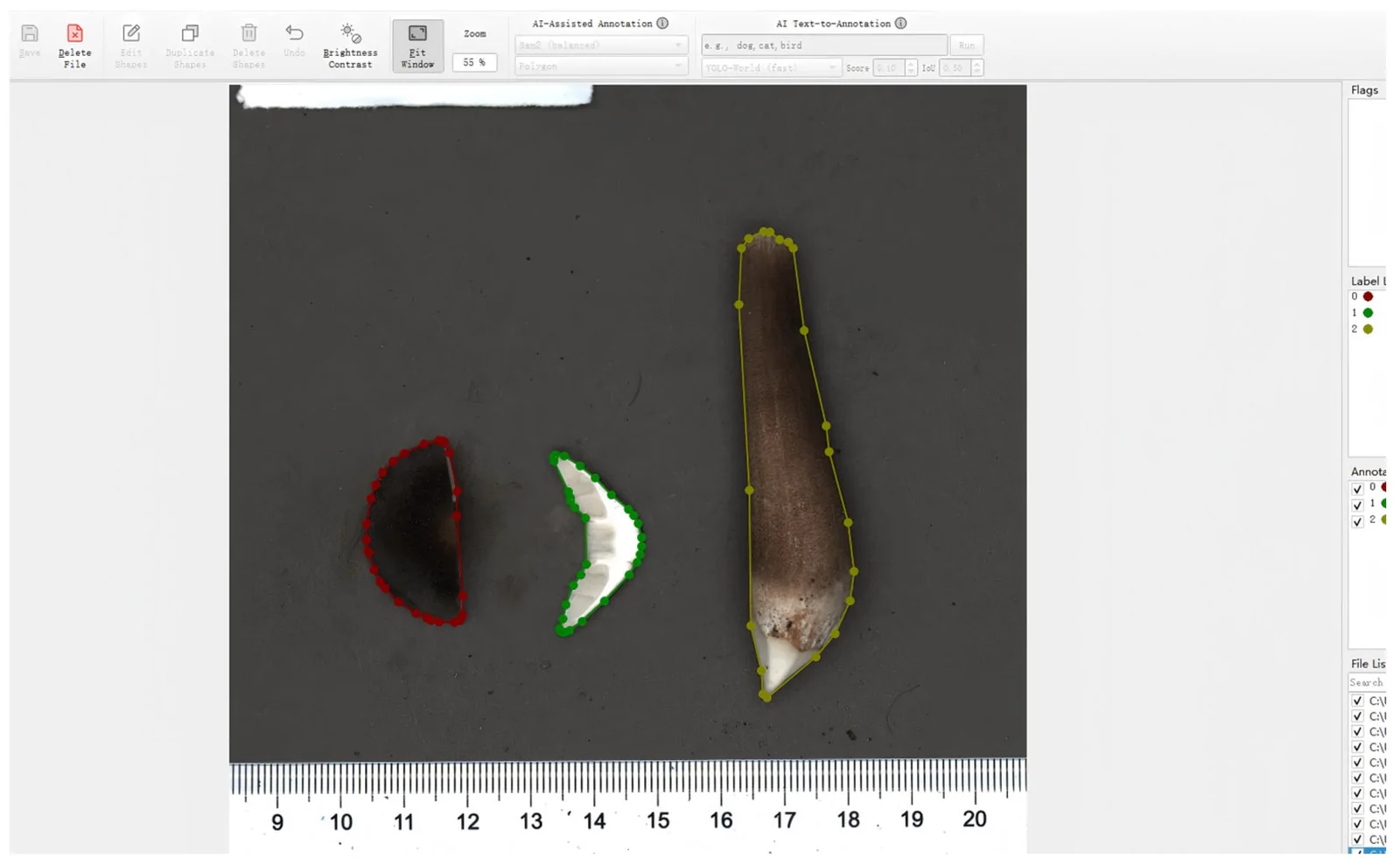
Data annotation image.

A total of 473 sample images were obtained, and the mushroom regions were manually annotated pixel by pixel using LabelMe (v6.3.1; Kentaro Wada, Tokyo, Japan), as shown in Figure 1, The red, green, and yellow dots represent the half pileus, cross-section of pileus, and stipe of O. raphanipes, respectively.yielding 1419 annotated mushroom objects. The YOLOv11s-star-seg (Ultralytics, v8.3.9, Frederick, MD, USA) model was trained using the official Ultralytics YOLO implementation. During training, images were resized to 1600 × 1600 pixels following the default Ultralytics YOLO letterbox preprocessing strategy, in which the original aspect ratio was preserved and the image was uniformly scaled before padding was added to obtain a square input size. The padding regions introduced during this automatic preprocessing procedure were filled using the default constant value of 114 for each RGB channel (RGB = 114, 114, 114). This padding value was determined by the YOLO preprocessing implementation and was not manually adjusted in this study. Therefore, the original 3:2 image aspect ratio was maintained during preprocessing, and no independent horizontal or vertical stretching was applied.

During inference, the original resolution images (5472 × 3648 pixels) were processed using the same YOLO letterbox preprocessing procedure required by the model. The predicted segmentation masks were subsequently mapped back to the original image coordinate system by reversing the letterbox transformation before phenotypic parameter extraction. Therefore, all morphological measurements, including pileus diameter, pileus thickness, stipe length, and stipe diameter, were calculated from segmentation masks restored to the original image resolution rather than from the resized network input images. The spatial resolution used for phenotypic measurement was therefore identical to the original acquisition resolution (5472 × 3648 pixels), and the padding regions introduced during model preprocessing did not contribute to quantitative measurements.

The pixel-to-length calibration coefficient was obtained using the geometric calibration grid captured under the same imaging conditions. The calibration coefficient was calculated based on the original image resolution (5472 × 3648 pixels) and was 0.00775 mm/pixel. This coefficient was then applied to convert pixel measurements from the restored original resolution masks into actual physical dimensions.

To maintain a similar distribution of the seven material types across the different subsets, a stratified splitting strategy based on material ID was adopted. For each of the seven materials (501, 504, 519, 525, 527, 528, and 531), the corresponding images were assigned to the training, validation, and test sets at an approximate ratio of 7:2:1, respectively. Consequently, all seven material types were represented in each subset, while each individual image and fruiting body appeared in only one subset, with no duplication among the training, validation, and test sets. The final training, validation, and test sets contained 331, 94, and 48 images, respectively. Cultivation batch was not used as an independent grouping factor during dataset partitioning; instead, images from different batches of the same material were pooled before stratified splitting. This strategy was adopted to maintain comparable material distributions among the three subsets.

### 2.2. Introduction to the Segmentation Model

The YOLOv11s-seg model follows the overall “end-to-end, one-stage detection” design concept of the YOLO series, performing object localization, category discrimination, and pixel-level mask prediction within the same network; it avoids the additional computational overhead caused by region proposal generation and secondary feature extraction in conventional two-stage methods such as Mask R-CNN, thereby structurally improving model real-time performance and ease of system integration. At the network architecture level, YOLOv11s-seg uses a lightweight backbone network as the feature-extraction module and enhances its representation of multiscale targets through progressive fusion of shallow and deep features. Shallow features focus on fine-grained information such as edges and textures and strongly represent detailed features such as crop leaf margins, panicle contours, and mechanical component boundaries; deep features focus on high-level semantic representation and facilitate effective differentiation between targets and backgrounds in complex field environments. In the neck network, a multiscale feature-fusion structure integrates features of different resolutions horizontally and vertically, enabling the model to maintain stable detection and segmentation performance for agricultural targets with substantial scale variation. YOLOv11s-seg achieves a relatively reasonable balance among model size, inference speed, and segmentation accuracy and is suitable for deployment in practical application scenarios such as agricultural robots, intelligent agricultural machinery, field monitoring terminals, and embedded vision systems. Compared with instance-segmentation models having larger parameter scales, it has lower computing power requirements and higher real-time response capability on edge-computing platforms, providing reliable visual algorithm support for real-time perception, precise operations, and intelligent decision-making in agricultural production.

### 2.3. Introduction to the Improved Segmentation Model

**Figure 2:**
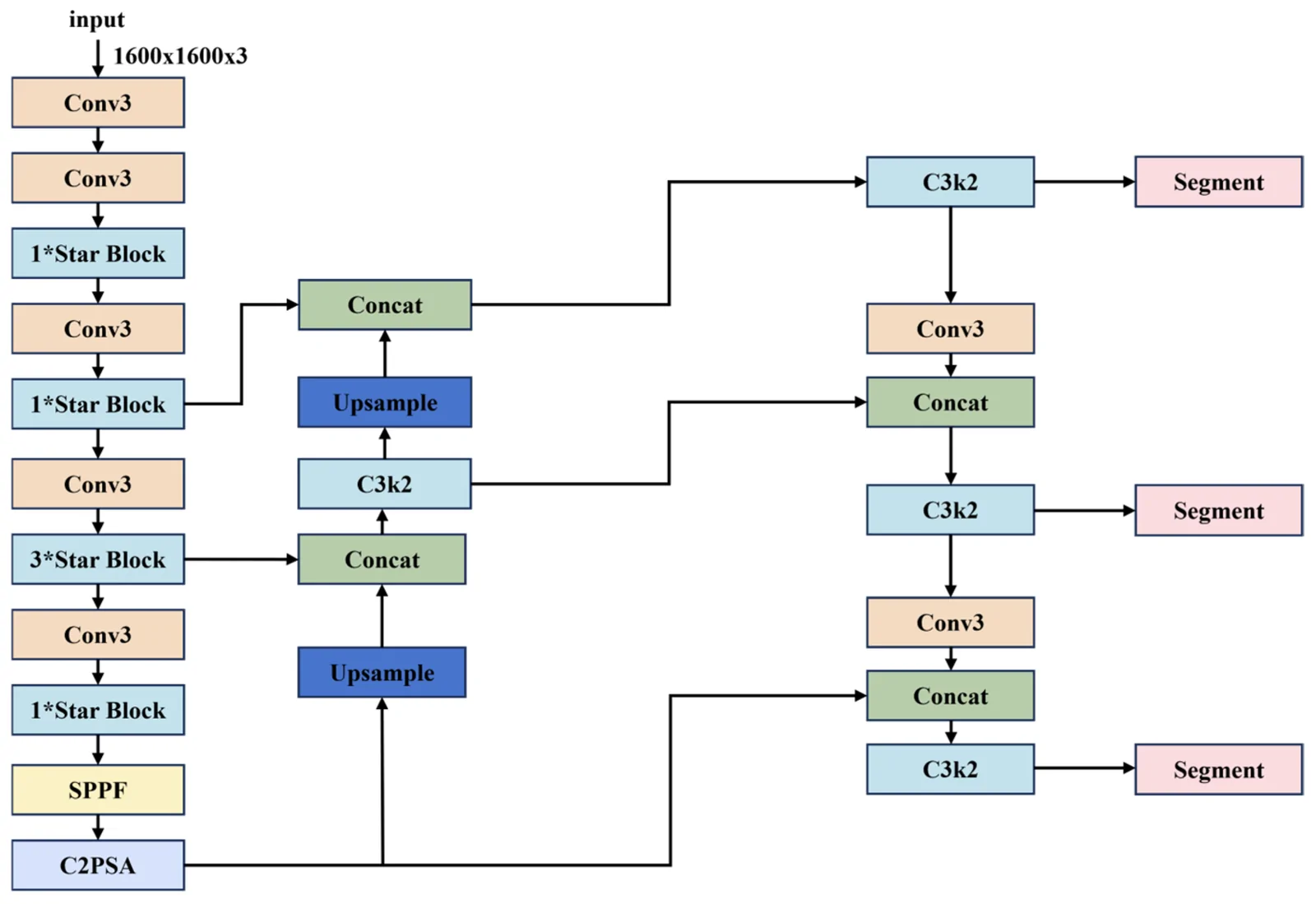
Architecture of the YOLOv11s-star-seg model.

To deploy a lightweight model in the imaging box for rapid acquisition of *O. raphanipes* phenotypic indicators, this study improved YOLOv11s-seg by lightweighting the backbone network and constructed an improved lightweight instance-segmentation model for *O. raphanipes*, YOLOv11s-star-seg, as shown in Figure 2.

In addition to the proposed StarNet module, other basic components of the network architecture are retained from the original YOLOv11s-seg framework. Specifically, C3k2 is a CSP-style feature-extraction module used in YOLOv11, which enhances gradient flow and feature representation through cross-stage partial connections while maintaining computational efficiency. The Conv3 module represents the convolutional operation used for feature transformation and dimensional adjustment in the network. The SPPF (Spatial Pyramid Pooling-Fast) module employs multiscale pooling operations to enlarge the receptive field and aggregate contextual information without significantly increasing computational cost. The C2PSA module is an attention-based feature-refinement module that enhances important feature responses by modeling spatial and channel-wise information.

In the proposed YOLOv11s-star-seg architecture, the C3k2, C2PSA, SPPF, and Conv3 modules located after each concatenation operation in the PAN neck are retained to refine the fused multiscale features before they are passed to the Segment head. Among these modules, SPPF and C2PSA maintain the original structures and functions of YOLOv11s-seg, while the main modification of this study focuses on replacing the original backbone feature-extraction components with the lightweight StarNet structure to reduce model complexity and computational cost.

#### Lightweight Feature-Extraction Network StarNet

StarNet is a convolutional neural network architecture constructed for lightweighting, and its backbone consists of four consecutive feature-extraction stages. Each stage consists of convolutional layers and Star Block units, and progressive abstraction and representation of features are achieved through hierarchical stacking. Compared with conventional convolutional structures, Star Block uses depthwise separable convolution (DWConv) internally to replace standard convolution operations, thereby reducing the number of parameters and computational complexity. At the same time, a channel shuffle mechanism is introduced to promote information interaction among channels and enhance feature representation capability while controlling computational overhead. As the network hierarchy deepens, the model can gradually transition from shallow texture information to high-level semantic information, thereby achieving multilevel feature modeling.

This study selected the lightweight StarNet_s050 model from the StarNet series as the feature-extraction backbone. The four stages of this model contain 1, 1, 3, and 1 Star Block units, respectively. The input image first passes through the Stem Layer for initial feature mapping; this layer consists of a convolutional layer, a batch normalization layer, and an activation function and is used to perform basic feature extraction and improve numerical stability during training. The feature maps then pass sequentially through Stage 1 to Stage 4, with each stage progressively increasing the channel dimension while performing downsampling, thereby completing spatial information compression and semantic information enhancement. The application of DWConv effectively reduces computational complexity, while the hierarchical stacking of Star Blocks ensures continuity and sufficiency in the feature-extraction process.

### 2.4. Definition and Extraction Methods for Phenotypic Traits

According to the NY/T 3715—2020 testing guideline, the following five traits were analyzed.

#### 2.4.1. Pileus Diameter

**Figure 3:**
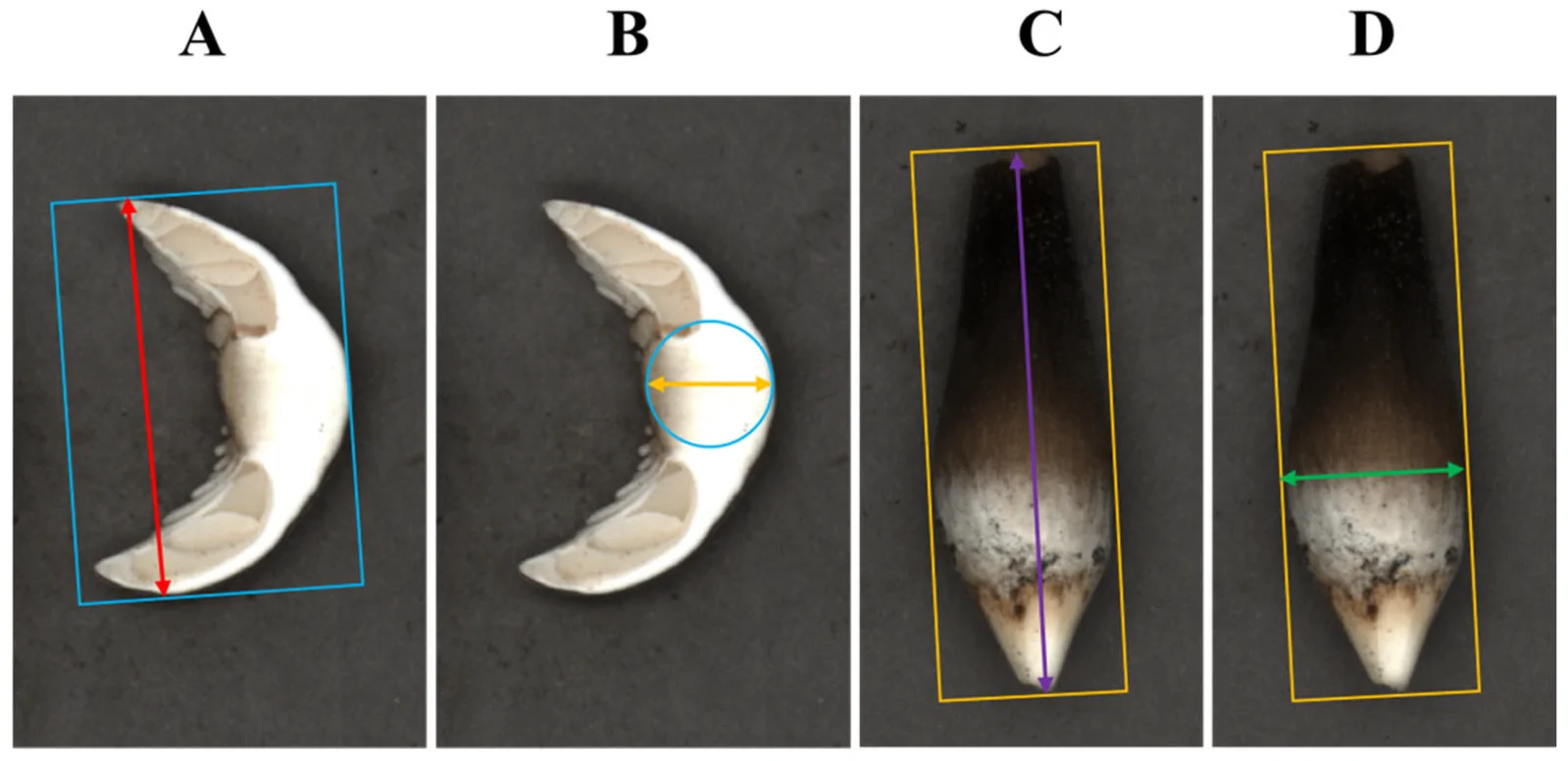
Schematic diagram of pileus and stipe phenotypes. Note: In Panel (**A**), the red arrow indicates the pileus diameter, which is obtained from the length of the minimum bounding rectangle (blue box). In Panel (**B**), the yellow arrow indicates the pileus flesh thickness, which is obtained from the diameter of the maximum inscribed circle (blue). In Panel (**C**), the purple arrow indicates the stipe length, which is obtained from the length of the minimum bounding rectangle (yellow). In Panel (**D**), the green arrow indicates the stipe diameter, which is obtained from the width of the minimum bounding rectangle (yellow).

The maximum transverse expansion of the pileus was defined as pileus diameter L1. The external contour was obtained from the pileus segmentation mask, the major axis length of the minimum bounding rectangle of the contour was calculated, and the value was converted to the actual size (mm) using the calibration coefficient, as shown in Figure 3A.

#### 2.4.2. Pileus Context Thickness

The thickness between the pileus margin and the pileus surface was defined as pileus context thickness. In the *O. raphanipes* image, the external contour was obtained from the pileus segmentation mask, the maximum inscribed circle of the contour was calculated, and the diameter of the maximum inscribed circle was taken as pileus context thickness L2 and converted to the actual size (mm) using the calibration coefficient, as shown in Figure 3B.

#### 2.4.3. Stipe Length

The longitudinal length from the bottom of the stipe to the junction with the pileus was defined as stipe length. The external contour was obtained from the stipe segmentation mask, the minimum bounding rectangle of the contour was calculated, and the major axis length of the minimum bounding rectangle was taken as stipe length L3 and converted to the actual size (mm) using the calibration coefficient, as shown in Figure 3C.

#### 2.4.4. Stipe Diameter

The transverse width of the stipe was defined as stipe diameter. The external contour was obtained from the stipe segmentation mask, the maximum inscribed circle of the contour was calculated, and the diameter of the maximum inscribed circle was taken as stipe diameter L4 and converted to the actual size (mm) using the calibration coefficient, as shown in Figure 3D.

#### 2.4.5. Stipe Shape

**Figure 4:**
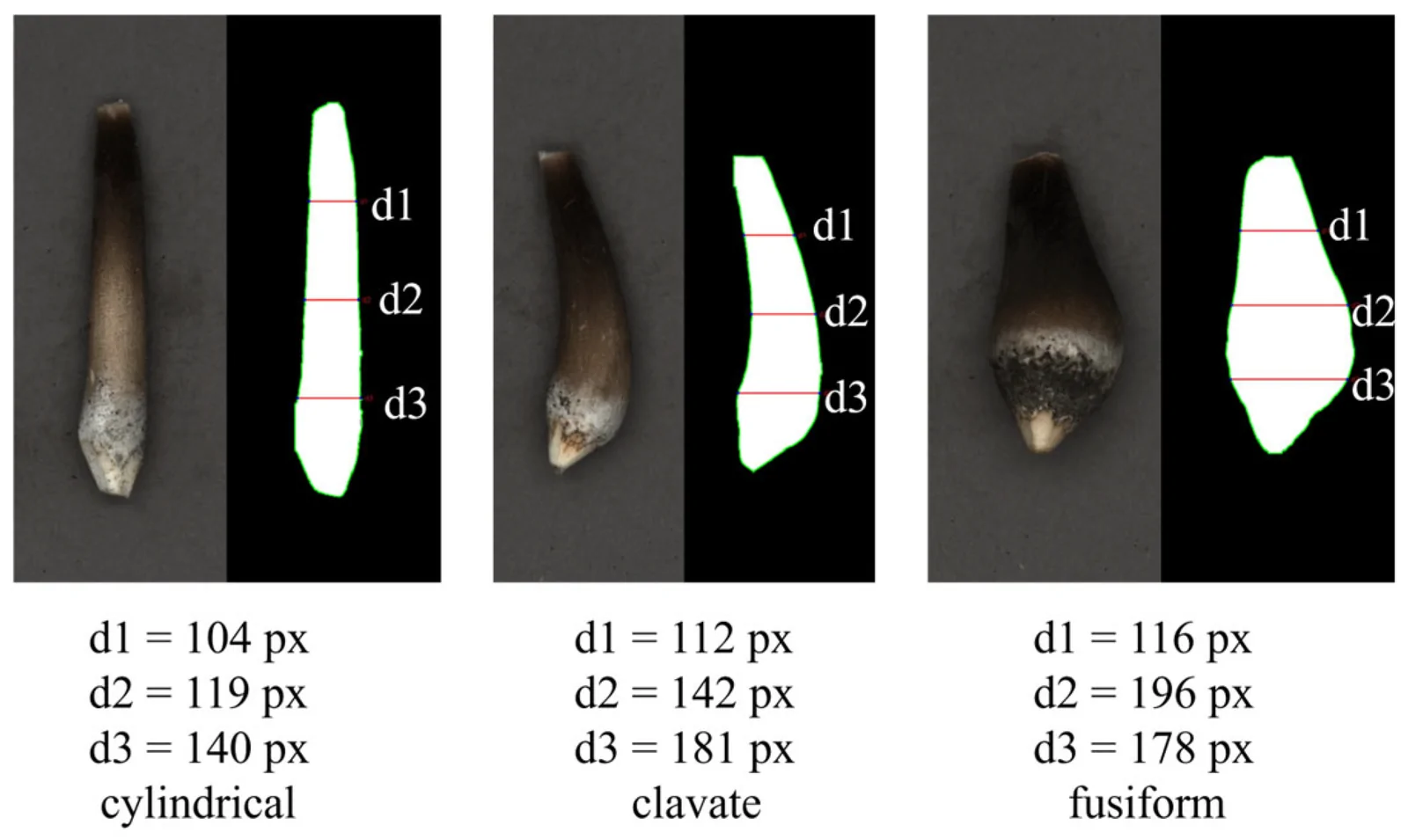
Diagram of stipe shape acquisition.

The overall morphological characteristics of the stipe included clavate, spindle-shaped, and cylindrical forms. The external contour was obtained from the stipe segmentation mask. Based on the stipe-contour region, its height was evenly divided into four segments along the longitudinal direction of the stipe, and three transverse measurement lines were constructed at one-quarter, two-quarters, and three-quarters of the height. The corresponding widths were denoted as d1, d2, and d3, respectively, as shown in Figure 4.

### 2.5. Model Training

All experiments and model training were conducted under the same hardware and software environment. In this study, the models were trained from scratch without loading pretrained weights. This strategy was adopted because the target task focuses on fine-grained instance segmentation of *Oudemansiella raphanipes*, whose morphological characteristics, including stipe structure, pileus shape, and boundary features, differ substantially from the natural image categories commonly used for pretrained models. Therefore, training from scratch was used to evaluate the feature-extraction capability of the proposed YOLOv11s-star-seg architecture under a consistent experimental setting and to avoid potential biases introduced by domain differences between pretraining datasets and mushroom images.

The training was implemented based on the PyTorch (v2.11.0; Meta AI, Menlo Park, CA, USA)deep learning framework. The experimental environment consisted of a Windows 11 operating system, NVIDIA GeForce RTX 3070 Ti GPU with driver version 31.0.15.5123, CUDA 11.8, and cuDNN 8.9.6.50. The training parameters were set as follows: BatchSize = 2, Workers = 2, Optimizer = SGD, Close-Mosaic = 10, Momentum = 0.937, and Learning Rate = 0.01. During model training, the input image size was set to 1600 × 1600 pixels.s, and Epochs = 300.

### 2.6. Model Performance Evaluation Metrics

To evaluate model performance, precision Pr (precision), recall R (recall), mean average precision (mAP), and mean intersection over union (mIoU) were used as evaluation metrics, and the calculation formulas were as follows:

(1) P_r_ = T_p_/(T_p_ + F_p_)

(2) R = T_p_/(T_p_ + F_n_)

(3) AP = ∫_0_^1^ P_r_(R) dR

(4) mAP = (1/C) ∑^c^_i=1_ AP_i_

(5) IoU = T_p_/(T_p_ + F_p_ + F_n_)

(6) mIoU = (1/C) ∑^c^_c=1_ IoU_c_

In Equations (1)–(6), Tp denotes a sample predicted as positive and actually positive, FP denotes a sample predicted as positive but actually negative, FN denotes a positive sample that was not predicted, C denotes the number of detected object categories, AP denotes average precision, and IoU measures the degree of overlap between the predicted and ground-truth regions.

## 3. Experimental Process

### Comparison of Model Results

After 300 training epochs, the YOLOv11s-star-seg algorithmic model was compared with the original YOLOv11s-seg model to verify the performance of YOLOv11s-star-seg.

The experimental results are shown in Table 2.

The proposed YOLOv11s-star-seg model achieved a precision (P) of 98.6%, a recall (R) of 99.3%, an mAP50 of 99.1%, an F1-score of 98.9%, and an mIoU of 97.86%. Compared with the original YOLOv11s-seg model, YOLOv11s-star-seg showed slight decreases in P and mAP50 (1.2 and 0.4 percentage points, respectively) while improving recall, F1-score, and mIoU. These results indicate that the proposed lightweight model maintained comparable segmentation performance while reducing model complexity.

**Figure 5:**
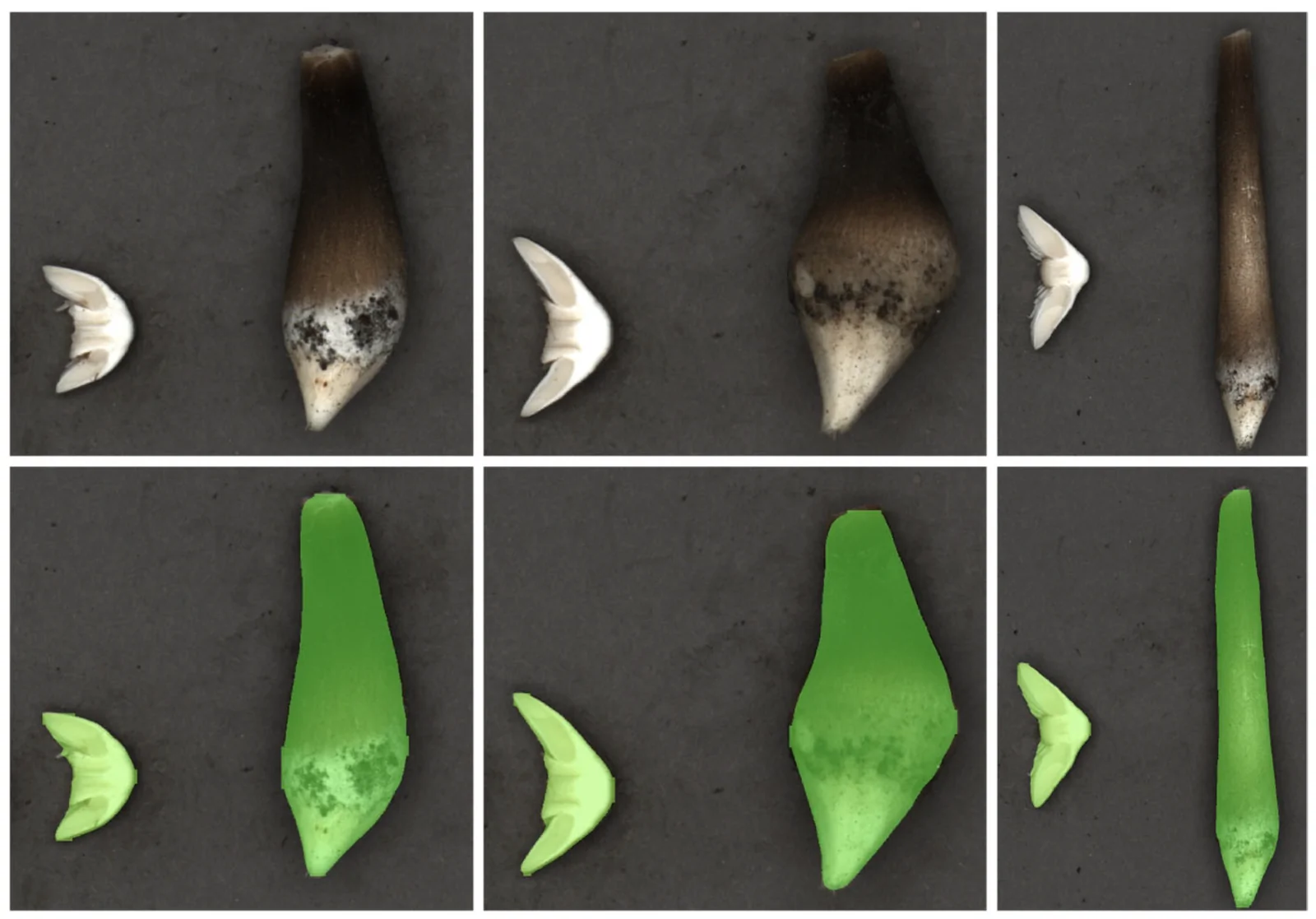
Recognition results of the YOLOv11s-star-seg model.

In terms of computational efficiency, the inference time of YOLOv11s-star-seg was reduced from 19.6 ms to 14.3 ms, representing a reduction of approximately 27.0%. Meanwhile, the number of parameters decreased from 10.08 M to 6.11 M, corresponding to a reduction of 39.4%. Therefore, the proposed YOLOv11s-star-seg model achieves a better balance between segmentation accuracy and computational efficiency, making it more suitable for deployment in the phenotyping imaging box. The segmentation results of *O. raphanipes* fruiting bodies are shown in Figure 5.

## 4. Results and Analysis

### 4.1. Verification of Model Improvement

After StarNet_s050 was used to replace the original YOLOv11s-seg backbone network, YOLOv11s-star-seg significantly reduced the number of model parameters and computational load while maintaining high instance-segmentation accuracy, mainly because StarNet balances feature-extraction capability and computational efficiency.

**Figure 6:**
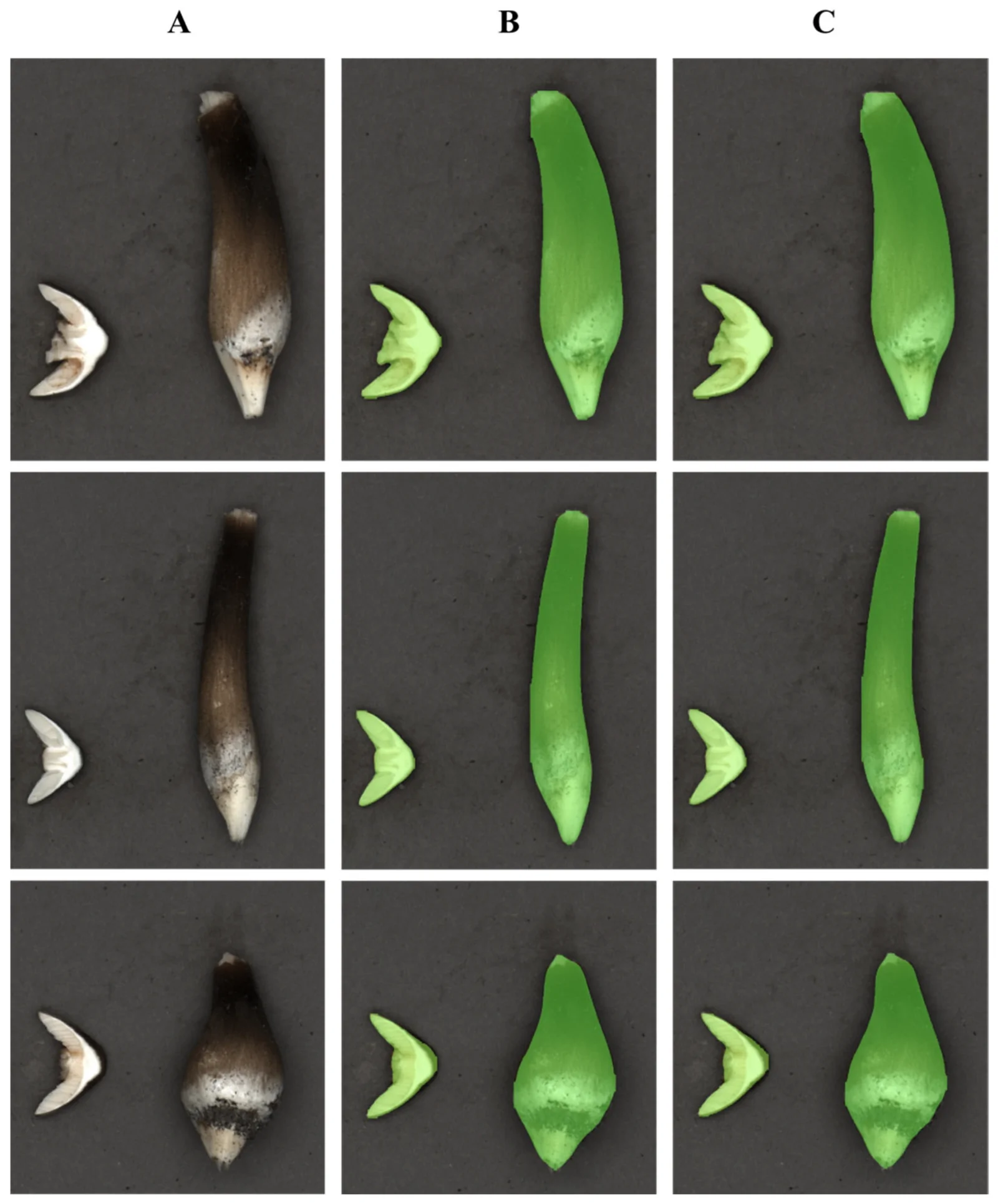
Comparison of model results. Column (**A**) shows the original *O. raphanipes* images; Column (**B**) shows the results of the YOLOv11s-seg model; and Column (**C**) shows the results of the YOLOv11s-star-seg model.

StarNet extensively uses depthwise separable convolution to replace conventional standard convolution, thereby effectively reducing model size and improving inference speed. While keeping network depth essentially unchanged, the model can complete feature-extraction tasks at a lower computational cost; therefore, its ability to extract key features such as *O. raphanipes* stipe contours, pileus edges, and target textures in complex backgrounds does not decrease significantly because of lightweighting, as shown in Figure 6.

### 4.2. Comparison Between Automatically Extracted Values and Manual Measurements

**Figure 7:**
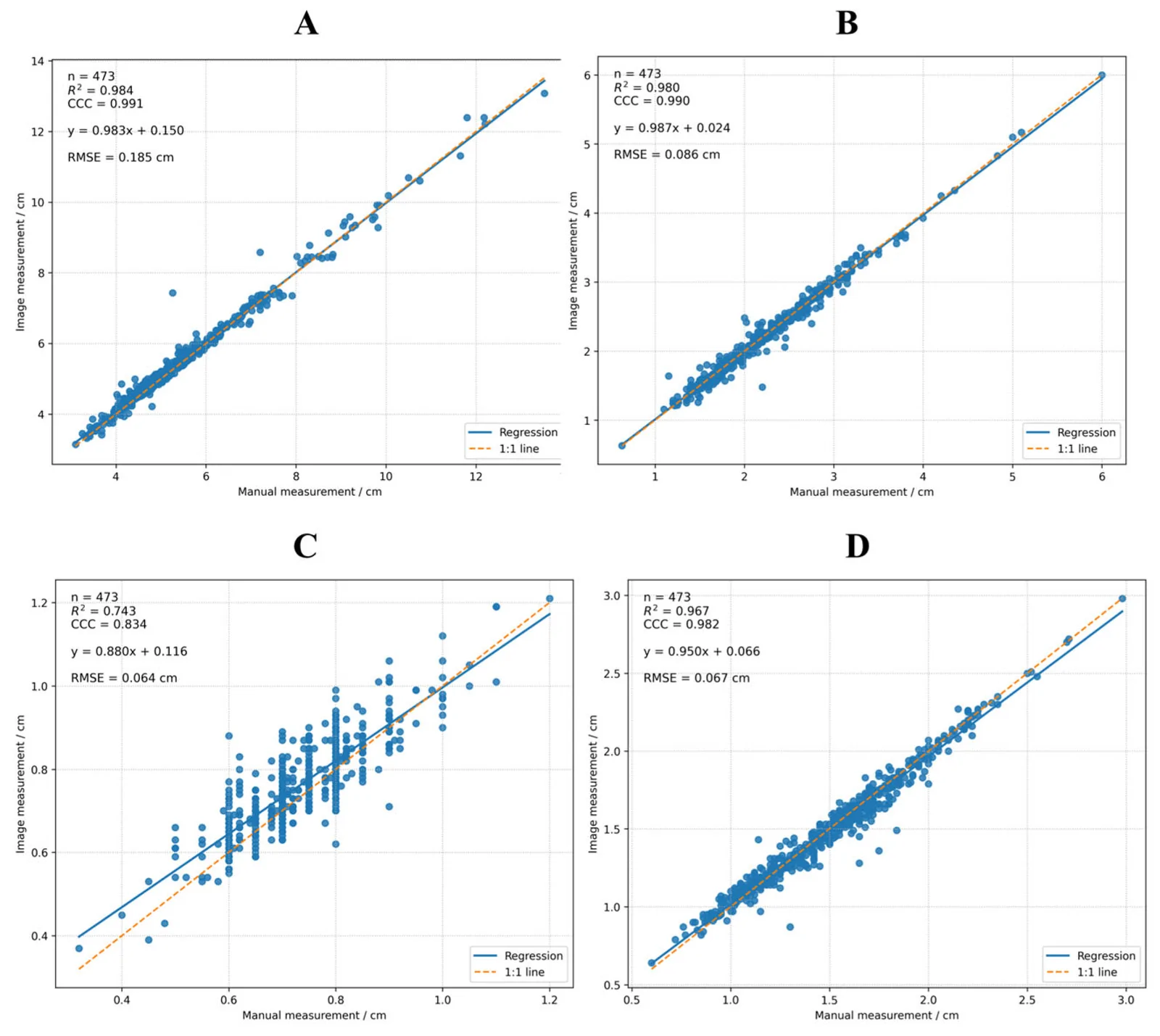
Linear regression analysis between automated measurements and manual measurements for four phenotypic traits of *Oudemansiella raphanipes*. Note: (**A**) Stipe length; (**B**) Pileus diameter; (**C**) Pileus flesh thickness; (**D**) Stipe diameter. The blue dots denote paired data values of automatic and manual measurements.

**Figure 8:**
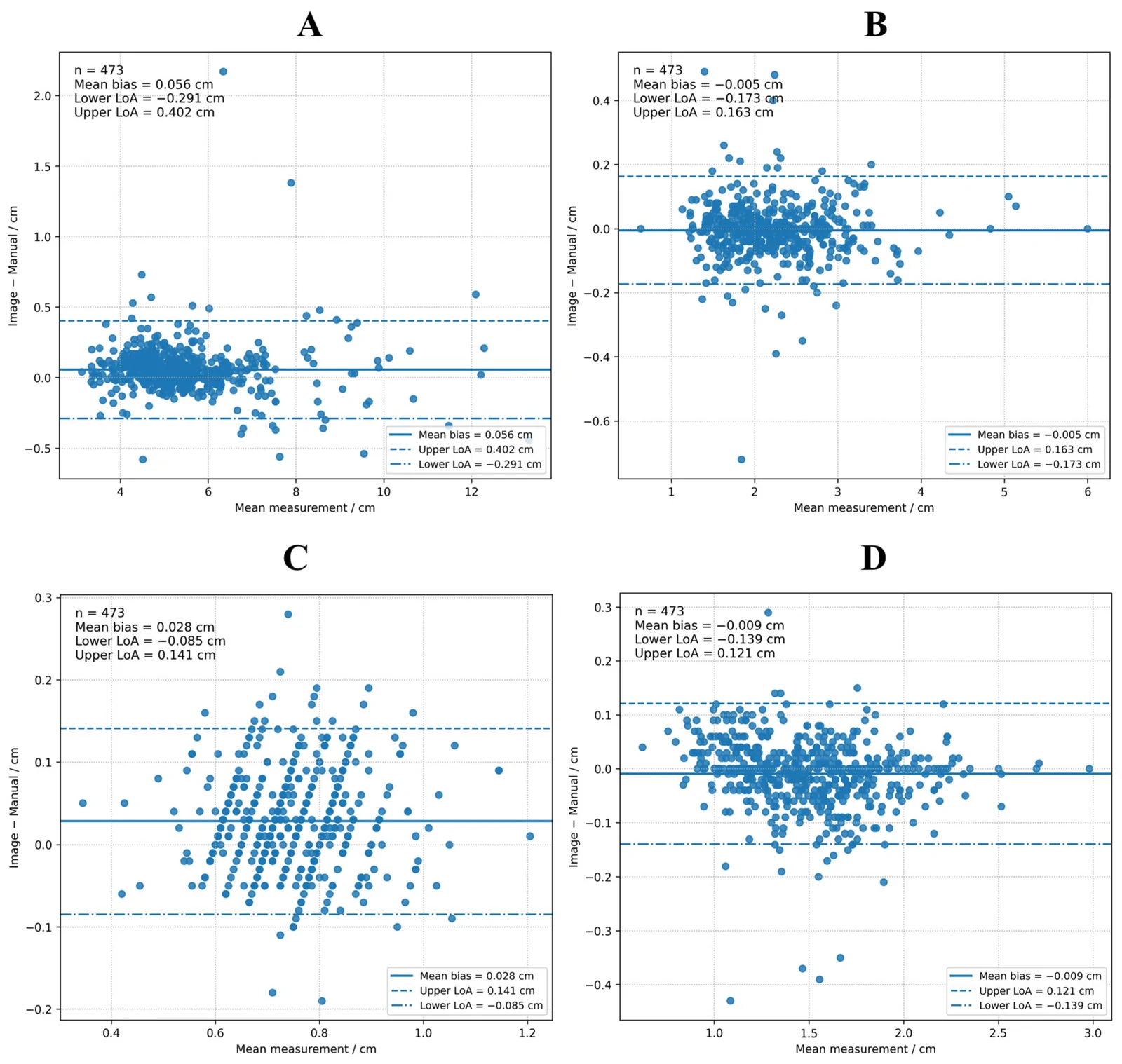
Bland–Altman analysis of agreement between automated measurements and manual measurements for four phenotypic traits of *Oudemansiella raphanipes*. Note: (**A**) Stipe length; (**B**) Pileus diameter; (**C**) Pileus flesh thickness; (**D**) Stipe diameter. The blue dots denote paired data values of automatic and manual measurements.

Linear regression analysis was performed between the measurements obtained using the proposed method and the manual measurements. As shown in Figure 7 and Figure 8, the automated measurements showed good agreement with the manual measurements. For stipe length (Figure 7A), pileus diameter (Figure 7B), and stipe diameter (Figure 7D), the coefficients of determination (R^2^) were all above 0.96, accompanied by high Lin’s concordance correlation coefficients (CCCs), indicating a high degree of consistency between the automated and manual measurements. The corresponding RMSE values also indicated relatively small measurement deviations, demonstrating the practical applicability of the proposed method for automated phenotypic measurement and its potential as an alternative to manual measurement in *O. raphanipes* DUS testing.

In contrast, the automatically extracted pileus thickness values (Figure 7C) showed relatively lower agreement with the manual measurements, with an R^2^ value of 0.742 and a CCC value of 0.834. The larger deviation may be associated with the difficulty of accurately characterizing pileus thickness from two-dimensional images and the difference between the image-derived geometric measurement and the manual measurement procedure. The possible causes are further discussed in the Discussion Section.

**Figure 9:**
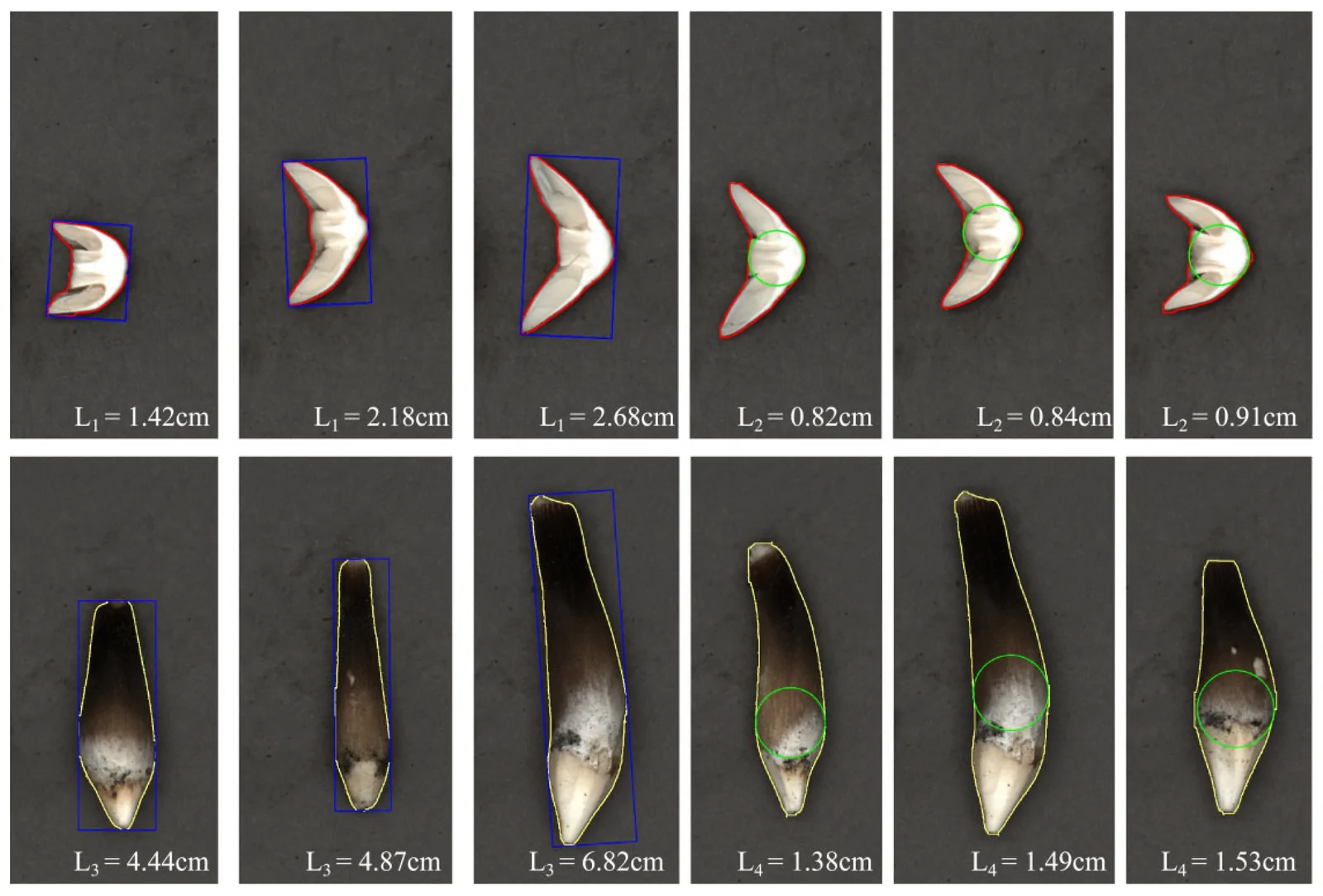
Automatically extracted results. Note: The blue boxes denote the minimum bounding rectangles of the pileus cross-section and stipe.The red line represents the contour of the pileus cross-section, and the yellow line represents the stipe contour.The green circles indicate the maximum inscribed circles of the pileus cross-section and stipe.L1is the pileus diameter, L2 is the pileus flesh thickness, L3 is the stipe length, and L4 is the stipe diameter.

Bland–Altman analysis was further performed to evaluate the agreement between the proposed method and manual measurements (Figure 8). The results showed that the biases of the measured traits were close to zero, and most measurements were distributed within the 95% limits of agreement (LoA), indicating no substantial systematic bias between the two measurement methods. The detailed statistical parameters, including sample number (*n*), R^2^, Lin’s CCC, slope, intercept, bias, and RMSE, are summarized in Table 3. The automatic measurement workflow and representative measurement results are shown in Figure 9.

### 4.3. Results of Stipe Shape Classification

To verify the effectiveness of the proposed stipe shape classification method, longitudinal width features were extracted from the stipe segmentation results and combined with the aspect ratio of the minimum bounding rectangle to characterize stipe morphology. Each stipe was evenly divided into four segments along its longitudinal axis, and the transverse widths (d1, d2, and d3) were measured at one-quarter, one-half, and three-quarters of the stipe height, respectively. These width measurements were used to characterize longitudinal variations in stipe shape.

To establish the reference labels of stipe shape, three industry experts with experience in edible mushroom DUS evaluation independently assessed the stipe morphology of all 473 samples. When the three experts reached complete agreement, the corresponding category was directly assigned as the reference label. When disagreements occurred, the final reference label was determined using majority voting. Expert disagreements mainly occurred for samples located in the transitional regions among fusiform, clavate, and cylindrical morphologies, where the boundaries between categories were visually ambiguous and difficult to distinguish solely by human observation.

Based on expert annotation, 81 samples were classified as fusiform; disagreements arose among experts for 15 of these samples. Among the remaining samples, 256 samples were classified as clavate, and 136 samples were classified as cylindrical, with expert disagreements occurring for 128 samples. Through the above multi-expert assessment and majority voting strategy, all 473 samples were assigned reference labels representing fusiform, clavate, and cylindrical stipe shapes, which were subsequently used as the reference standard for evaluating the rule-based classification method.

The classification thresholds were established by analyzing the longitudinal width distribution characteristics of the 473 samples with expert-defined reference labels. According to the observed morphological patterns, a stipe was classified as fusiform when (d2 > 1.05d1) and (d2 > 1.05d3), reflecting characteristic expansion in the middle region. A stipe was classified as clavate when (d3 > 1.55d1), indicating pronounced enlargement toward the lower region. Samples that did not satisfy either criterion were classified as cylindrical, corresponding to relatively small longitudinal width variation and an approximately uniform stipe profile. After determination, these thresholds were fixed and applied consistently to all 473 samples. The resulting classification performance is presented in Table 4.

As shown in the table, the classification accuracies for clavate, cylindrical, and spindle-shaped stipes were 76.17%, 77.21%, and 98.77%, respectively.

## 5. Discussion

This study addressed the time- and labor-intensive manual acquisition of edible mushroom fruiting body phenotypes, particularly the low measurement efficiency, high randomness, and insufficient repeatability associated with DUS testing of small umbrella-shaped edible mushrooms such as *O. raphanipes*. Seven *O. raphanipes* materials and a total of 473 images were used. An instance-segmentation model improved from YOLOv11s-seg reduced model complexity while maintaining comparable segmentation performance and, in combination with image-processing methods, enabled automatic extraction of multiple morphological traits. During DUS trait measurement, the extraction of stipe length, pileus diameter, and other morphological parameters depends strongly on accurate target contours [32]. Segmentation errors can propagate to subsequent image-processing steps and thereby affect agreement with manual measurements. The automated workflow established by combining instance segmentation with image-processing algorithms can reduce manual involvement, improve testing efficiency and standardization, and provide technical support for the digitalization of DUS testing.

Comparison between automatically extracted and manually measured values showed relatively high agreement for stipe length, stipe diameter, and pileus diameter, with R^2^ values of 0.98, 0.96, and 0.98, respectively. In contrast, pileus thickness showed lower agreement, with an R^2^ value of 0.74. For stipe shape discrimination, the classification accuracy for the fusiform type was relatively high at 98.77%, whereas the accuracies for the clavate and cylindrical types were lower, at 76.17% and 77.20%, respectively.

The relatively large discrepancy observed for pileus thickness may arise from both manual measurement uncertainty and the definition of the image-derived thickness indicator. In this study, pileus thickness was estimated from a two-dimensional geometric feature based on the diameter of the maximum inscribed circle extracted from the image. This image-based parameter represents a geometric surrogate rather than the actual physical thickness measured according to the DUS procedure. Theoretically, the diameter of the maximum inscribed circle is smaller than the actual pileus thickness, and when the thickness value is relatively small, the relative error introduced by this geometric approximation becomes more pronounced compared with larger traits such as stipe length and stipe diameter. In addition, pileus thickness in *O. raphanipes* is generally only 2–5 mm, and the pileus tissue is relatively soft. During manual measurement, contact pressure from the vernier caliper may cause slight deformation, while operator-dependent positioning may introduce additional variation. Therefore, the observed discrepancy should be interpreted as the combined result of differences between measurement definitions, limitations of two-dimensional image-based estimation, and uncertainty associated with manual measurement. The current findings also highlight the need for improved image-based algorithms or three-dimensional measurement approaches for more accurate pileus thickness estimation in future studies.

For stipe shape classification, the DUS testing guideline for *O. raphanipes* provides representative illustrations of three stipe shape categories, including fusiform, clavate, and cylindrical types, but does not provide explicit quantitative definitions. Based on discussions with edible mushroom experts, preliminary morphological criteria were established in this study: fusiform stipes are characterized by a thicker middle region and thinner upper and lower regions; clavate stipes show a pronounced enlargement toward the lower region with a relatively narrower upper part; and cylindrical stipes exhibit relatively uniform upper and lower widths without an obvious lower-expansion characteristic. Based on these morphological characteristics, fusiform stipes showed clear differences from both clavate and cylindrical forms, which contributed to the relatively high classification accuracy (98.77%). In contrast, clavate and cylindrical stipes exhibited greater similarity in longitudinal width distribution patterns, with their main difference reflected in the relative width relationship between the upper and lower regions of the stipe. Therefore, classification based only on the ratio between d3 and d1 remains insufficient for accurately distinguishing these two morphologies. Additional geometric descriptors and more comprehensive shape features should be incorporated to improve discrimination between clavate and cylindrical stipes.

Although this study achieved satisfactory segmentation and phenotypic measurement performance, several limitations remain. First, the model training and test data were mainly obtained under a relatively fixed imaging environment, and the model’s generalization ability under different lighting conditions, background materials, cultivation environments, and a wider range of varieties has not yet been fully verified [33]. Second, although all models in this study were trained from scratch under the same experimental conditions to facilitate a consistent comparison of network architectures, the dataset was relatively small, and no direct comparison between training from scratch and transfer learning using pretrained weights was conducted. Therefore, the present results should not be interpreted as demonstrating that training from scratch is superior to transfer learning. Pretrained models may improve convergence and generalization under limited data conditions, and this should be systematically evaluated in future work. Third, additional parameter variables and shape descriptors are required for the definition of stipe morphology to improve discrimination between cylindrical and clavate forms. Fourth, although the model was optimized for lightweight deployment, further compression may still be required for real-time processing of large-scale data or deployment on embedded devices. Fifth, because the pilei and stipes of the samples used in this study were predominantly dark brown and did not adequately cover other color phenotypes, such as light brown, grayish brown, and medium brown, quantitative classification of pileus and stipe color could not be fully evaluated.

Future research should therefore focus on several aspects. First, multi-environment, multi-batch, and multi-variety sample data should be expanded to improve model robustness and generalization across different DUS testing scenarios [34]. Second, three-dimensional information obtained using depth estimation, stereo vision, or multiview imaging could be incorporated to improve the measurement of structural parameters such as pileus thickness and to reduce the limitations inherent in two-dimensional geometric surrogates [35]. Third, transfer learning strategies should be systematically compared with training from scratch to evaluate their effects on convergence, accuracy, and generalization under limited data conditions. Finally, additional lightweight strategies, including model pruning and quantization, could be explored to further improve deployment efficiency on low-computing-power devices.

## Figures and Tables

**Table 1:** Frequency distribution table.

Variety	501	504	519	525	527	528	531
Quantity	67	67	68	68	67	68	68

**Table 2:** Comparison of model results.

Model	P (%)	R (%)	mAP50 (%)	F1 (%)	mIoU (%)	Inference Time (ms)	Params (M)
YOLOv11s-seg	99.8	97.2	99.5	98.5	97.04	19.6	10.08
YOLOv11s-star-seg	98.6	99.3	99.1	98.9	97.86	14.3	6.11

**Table 3:** Statistical comparison between automated measurements and manual measurements for different phenotypic traits of *O. raphanipes*.

**Category**	**Quantity**	**R^2^**	**Lin’s CCC**	**Slope**	**Intercept**	**Bias (cm)**	**RMSE (cm)**
Stipe length	473	0.984	0.991	0.982	0.149	0.055	0.185
Stipe diameter	473	0.966	0.982	0.949	0.066	−0.009	0.066
Pileus thickness	473	0.742	0.834	0.880	0.116	0.028	0.063
Pileus diameter	473	0.980	0.989	0.986	0.024	−0.005	0.085

**Table 4:** Classification accuracy for each morphological category.

Shape Category	Total	Correct	Incorrect	Accuracy (%)
Clavate	256	195	61	76.17
Cylindrical	136	105	31	77.21
Spindle-shaped	81	80	1	98.77

## Data Availability

The datasets used and/or analyzed during the current study are available from the corresponding author on reasonable request.
